# Multivariate Analysis of Anthropometric Traits Using Summary Statistics of Genome-Wide Association Studies from GIANT Consortium

**DOI:** 10.1371/journal.pone.0163912

**Published:** 2016-10-04

**Authors:** Haeil Park, Xiaoyin Li, Yeunjoo E. Song, Karen Y. He, Xiaofeng Zhu

**Affiliations:** 1 Department of Epidemiology and Biostatistics, School of Medicine, Case Western Reserve University, Cleveland, OH, 44106, United States of America; 2 Department of Laboratory Medicine, Bucheon St. Mary’s Hospital, College of Medicine, The Catholic University of Korea, Seoul, Republic of Korea; University of the Chinese Academy of Sciences, CHINA

## Abstract

Meta-analysis of single trait for multiple cohorts has been used for increasing statistical power in genome-wide association studies (GWASs). Although hundreds of variants have been identified by GWAS, these variants only explain a small fraction of phenotypic variation. Cross-phenotype association analysis (CPASSOC) can further improve statistical power by searching for variants that contribute to multiple traits, which is often relevant to pleiotropy. In this study, we performed CPASSOC analysis on the summary statistics from the Genetic Investigation of ANthropometric Traits (GIANT) consortium using a novel method recently developed by our group. Sex-specific meta-analysis data for height, body mass index (BMI), and waist-to-hip ratio adjusted for BMI (WHRadjBMI) from discovery phase of the GIANT consortium study were combined using CPASSOC for each trait as well as 3 traits together. The conventional meta-analysis results from the discovery phase data of GIANT consortium studies were used to compare with that from CPASSOC analysis. The CPASSOC analysis was able to identify 17 loci associated with anthropometric traits that were missed by conventional meta-analysis. Among these loci, 16 have been reported in literature by including additional samples and 1 is novel. We also demonstrated that CPASSOC is able to detect pleiotropic effects when analyzing multiple traits.

## Introduction

For over a decade, genome-wide association studies (GWASs) have been a major tool for detecting genetic variants underlying complex traits [1], including various anthropometric traits. Anthropometric traits such as height and body mass index (BMI) are highly heritable [2, 3], but the genetic loci that have been reported by large GWASs so far only account for a small portion of heritability, suggesting additional variants still need to be uncovered. Up until now, most GWASs were performed by examining a single trait association, although it has been frequently observed that the same variant or gene can be associated with multiple traits [4]. During the past decade, the number of meta-analysis of GWASs using multiple cohorts has increased in order to maximize the statistical power in detecting significant genetic loci associated with a trait [5]. Several large-scale meta-analyses have been conducted for anthropometric traits, such as height, BMI, and waist-to-hip ratio (WHR) [6–11]. Among these three anthropometric traits, height is a classic polygenic trait that has been heavily studied in order to understand the genetic architecture of complex traits or diseases [9]. BMI is a convenient measure of overall adiposity as well as obesity, which is a risk factor for many diseases. WHR is a measure of body fat distribution and WHR adjusted for BMI (WHRadjBMI) is positively associated with mortality. Up until recently, 423, 97, and 49 loci have been identified to be associated with height, BMI, and WHRadjBMI in European populations, respectively [9–11].

Several multivariate analysis approaches for analyzing multiple phenotypes have been developed to further improve statistical power and provide biological interpretation of results, and their pros and cons were well reviewed [4]. Statistical methods of integrating evidence from summary statistics of multiple traits have recently been developed [12–14]. In traditional meta-analysis studies, the samples from different cohorts are assumed to be independent. Overlapped or related samples between different cohorts and correlated traits within the same cohorts would result in correlation among effect sizes, and both would lead to an inflated type I error rate. However, this issue can be resolved by estimating the correlation of effect sizes using summary statistics from genetic markers across the genome [12, 13, 15]. Solovieff et al. [4] coined the association of a genetic variant with multiple traits as cross-phenotype (CP) association. Zhu et al. demonstrated that power could be improved by testing multiple traits using both simulated and real data [12, 16]. CP association implies potential pleiotropy where a variant is associated with multiple traits regardless of underlying causes. Thus, CP association is more general than pleiotropy. Statistical software package for CP association analysis (CPASSOC) was developed to meta-analyze association evidence of genetic variant with correlated traits [12].

In this paper, we hypothesized that genetic variants commonly underlying the three anthropometric traits can be identified by combining all three traits when single trait analyses may not have enough power to detect them. Since the three traits are correlated, conventional meta-analysis assuming independence will result in an inflated type I error. In addition, it is unclear whether there are any overlapped or related samples among GIANT consortium studies, which would lead to correlated summary statistics among cohorts. Findings from meta-analysis accounting for correlation among cohorts will result in a more accurate estimation of genetic effects on traits. In this study, we compared the results of CPASSOC with that from conventional meta-analysis of GIANT consortium studies when using the same discovery phase data set. We performed meta-analysis with summary statistics of height, BMI, and WHRadjBMI obtained from the GIANT consortium [17] using the CPASSOC software.

## Results

**Fig 1:**
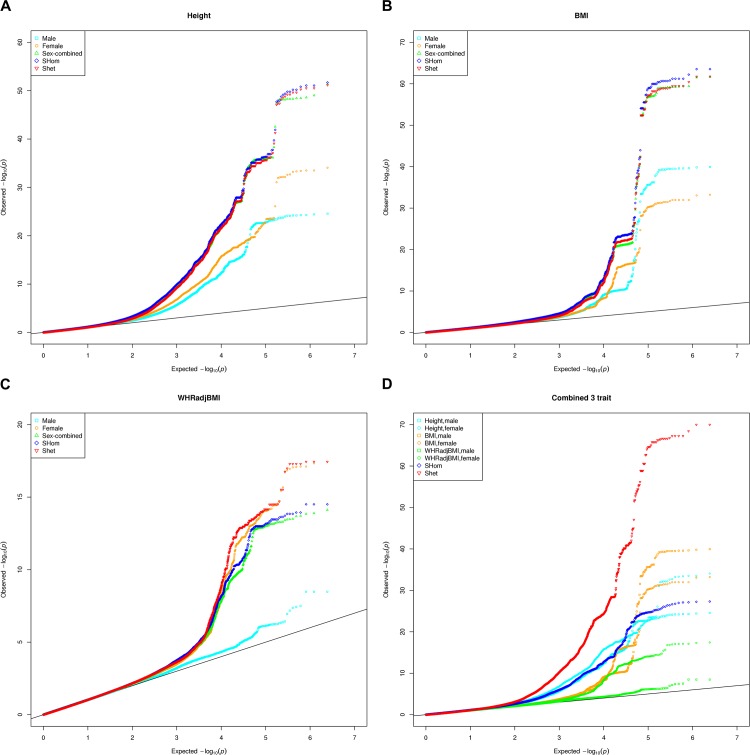
Q-Q plots of GIANT discovery result, CPASSOC *S*_*Hom*_ and *S*_*Het*._ (A) Height. (B) BMI. (C) WHRadjBMI. (D) Combining three traits.

GIANT consortium provided sex-specific summary statistics for the three traits: height, BMI and WHRadjBMI [17], which were downloaded from the GIANT consortium website (https://www.broadinstitute.org/collaboration/giant/index.php/GIANT_consortium_data_files). The correlations among the six cohorts were calculated using the summary statistics of common SNPs after linkage disequilibrium pruning (Materials and Methods, S1 Table). The correlation between male and female summary statistics is 0.03, 0.096 and 0.0126 for WHRadjBMI, BMI and height, respectively (S2 Table). The correlations for BMI and height are still high despite of excluding the SNPs with absolute Z score larger than 1.96. The large correlations between male and female summary statistics for height and BMI suggest that many genetic variants with small effect sizes contribute both trait variations besides the possible overlapped or related samples. The correlation was much smaller between traits than within traits (S2 Table). We next calculated statistics *S*_*Hom*_ and *S*_*Het*_ in CPASSOC (Materials and Methods). *S*_*Hom*_ makes an assumption that genetic effect is homogenous across traits and cohorts while *S*_*Het*_ assumes genetic heterogeneity. Fig 1 presents the Q-Q plots for height, BMI, and WHRadjBMI individually as well as combined together, using *S*_*Hom*_ and *S*_*Het*_. The genomic control values (λ) for statistics S_*Hom*_, are 1.078, 1.079, 1.025 and 1.092 for height, BMI, WHRadjBMI and combining the three traits, respectively. For S_*Het*_, the λ values are 0.998, 1.006, 0.994 and 0.992 for height, BMI, WHRadjBMI and combining three traits, respectively. We did not observe any inflation for *S*_*Het*_ but slight inflation was observed for height and BMI for *S*_*Hom*_. Both height and BMI have many causal variants, which can inflate the corresponding λ values. In the original and the latest GIANT reports [18, 19], the λ values were 1.42 for height and 1.526 for BMI. Our observed λ values for both *S*_*Hom*_ and *S*_*Het*_ are much smaller than that in GIANT reports. Thus, our observed association evidence is unlikely due to the effects of population stratification. We observed that females have substantially more genetic contribution than males for WHRadjBMI (Fig 1C). Table 1 compares the number of significant loci (P < 5 × 10^−8^) detected by CPASSOC and conventional meta-analysis by GIANT. For height, both conventional meta-analysis and *S*_*Hom*_ identified 116 loci and 3 of them were missed by either method, while *S*_*Het*_ identified 89 loci. For BMI, *S*_*Hom*_ and *S*_*Het*_ identified 20 and 17 loci, respectively, while the conventional meta-analysis detected 18 loci. For WHRadjBMI, *S*_*Hom*_ and *S*_*Het*_ identified 11 and 14 loci, respectively, while meta-analysis detected 11 loci. The detailed P-values and effect sizes for these variants in CPASSOC and conventional meta-analysis are presented in S3 Table. P-values for combining the three traits, *S*_*Hom*_ and *S*_*Het*_ identified 55 and 129 loci, respectively, while conventional meta-analysis identified 122 loci for three traits together. The detailed P-values and effect sizes for these variants in CPASSOC and conventional meta-analysis are presented in S4 Table. The reason for substantially fewer variants detected by S_*Hom*_ is due to different effect directions in the three traits.

**Table 1:** The number of genome-wide significant loci identified by CPASSOC for height, BMI, and WHRadjBMI from sex-specific data of discovery phase.

			GIANT Consortium Studies^b^		
Trait	CPASSOC Method^a^		P < 5 × 10^−8^	P > 5 × 10^−8^	Total
Height	*S*_*Hom*_	P < 5 × 10^−8^	113	3	116
		P > 5 × 10^−8^	3		3
		Total	116	3	
	*S*_*Het*_	P < 5 × 10^−8^	89	0	89
		P > 5 × 10^−8^	27		27
		Total	116	0	
BMI	*S*_*Hom*_	P < 5 × 10^−8^	17	3	20
		P > 5 × 10^−8^	1		1
		Total	18	3	
	*S*_*Het*_	P < 5 × 10^−8^	16	1	17
		P > 5 × 10^−8^	2		2
		Total	18	1	
WHRadjBMI	*S*_*Hom*_	P < 5 × 10^−8^	10	1	11
		P > 5 × 10^−8^	1		1
		Total	11	1	
	*S*_*Het*_	P < 5 × 10^−8^	11	3	14
		P > 5 × 10^−8^	0		0
		Total	11	3	

Note: CPASSOC (cross-phenotype association), GIANT (genetic investigation of anthropometric traits), BMI (body mass index), WHRadjBMI (waist-to-hip ratio adjusted for body mass index)

^a^CPASSOC was applied to meta-analyze male and female data for each of the three traits.

^b^The result of conventional meta-analyses of discovery phase data for each of the three traits.

**Fig 2:**
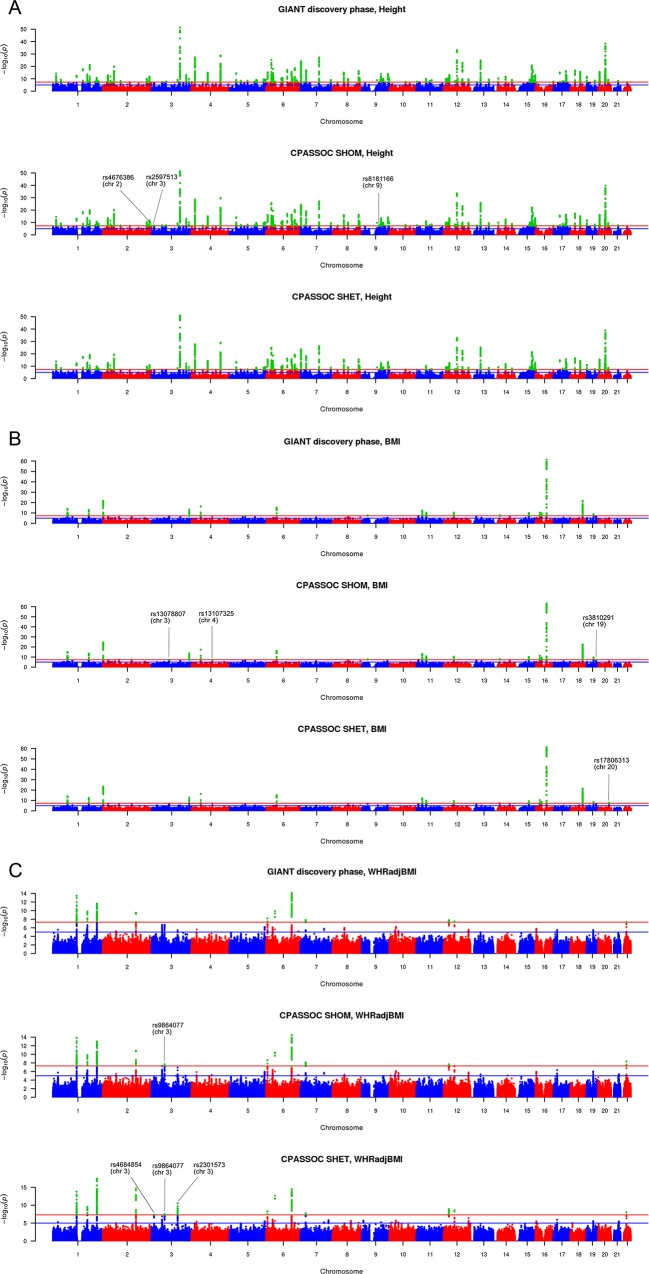
Manhattan plots of conventional meta-analysis, *S*_*Hom*_ and *S*_*Het*_ for three traits. The loci reaching genome-wide significance by *S*_*Hom*_ and *S*_*Het*_ but not by conventional meta-analysis are marked with corresponding SNP names. (A) Height by conventional meta-analysis, *S*_*Hom*_ and *S*_*Het*_; (B) BMI by conventional meta-analysis, *S*_*Hom*_ and *S*_*Het*_; (C) WHRadjBMI by conventional meta-analysis, *S*_*Hom*_ and *S*_*Het*_.

The Manhattan plots for the trait specific analysis are presented in Fig 2. The three loci identified by *S*_*Hom*_ but not by conventional meta-analysis for height are rs4676386, rs2597513, and rs8181166 (Table 2 and Fig 2A). Variants rs2597513 and rs8181166 were reported to be associated with height in meta-analyses combining discovery and follow-up phases [6], which had a substantially increased sample size. The region of rs4676386 has been reported in [9], and the reported variant rs4344931 was located 43,541 bp away from rs4676386. Those two variants were in weak linkage disequilibrium (r^2^ = 0.18).

**Table 2:** Additional genome-wide significant loci identified by CPASSOC for each trait but not conventional meta-analysis of GIANT consortium.

						CPASSOC		Conventional Meta-analysis of GIANT consortium	Sex-specific Meta-analysis of GIANT consortium					
						SHOM	SHET		Male			Female		
	SNP	Chr	Position (bp)	Effect / other allele	Frequency (effect allele)	*P*-value	*P*-value	P-value	Beta (Males)	SE (Males)	*P*-value (Males)	Beta (Females)	SE (Females)	*P*-value (Females)
Height	rs4676386	2	240835569	A/T	0.44	4..19E-08	2.16E-7	3.10E-07	0.022	0.0063	5.90E-03	0.027	0.0058	4.99E-06
	rs2597513	3	13514336	T/C	0.88	2.51E-08	1.30E-07	1.14E-07	-0.041	0.02	3.88E-05	-0.04	0.0093	2.11E-05
	rs8181166	9	86501713	C/G	0.52	1.73E-08	9.04E-08	1.09E-07	0.017	0.063	6.3E-03	0.032	0.058	7.21E-08
BMI	rs13078807	3	85835000	A/G	0.82	3.77E-08	2.65E-07	1.06E-07	-0.025	0.0079	1.4E-03	-0.037	0.0076	1.22E-06
	rs13107325	4	102267552	T/C	0.12	3.15E-08	2.22E-07	1.37E-07	0.062	0.014	1.1E-05	0.054	0.014	1.6E-04
	rs3810291	19	47065746	A/G	0.63	4.13E-08	2.89E-07	1.4E-07	0.031	0.0073	2.59E-05	0.028	0.0071	7.46E-05
	rs17806313	20	52478024	A/G	0.73	1.65E-06	1.83E-08	1.07E-06	0.0048	0.007	0.49	0.04	0.0067	3.5E-09
WHRadjBMI	rs9864077	3	64719215	T/C	0.76	1.96E-08	4.62E-08	1.85E-07	0.018	0.0073	0.016	0.038	0.0071	1.06E-07
	rs4684854	3	12447383	C/G	0.43	1.3E-05	4.13E-08	1E-04	4E-04	0.0071	0.96	0.04	0.0071	2.36E-08
	rs2301573	3	129587076	T/C	0.93	1.21E-07	2.88E-11	3.68E-06	0.0025	0.012	0.83	0.074	0.022	9.33E-11

For BMI, *S*_*Hom*_ identified rs13078807, rs13107325, and rs3810291 and *S*_*Het*_ identified rs17806313, all of them were missed by conventional meta-analysis (Table 2 and Fig 2B). Among these four variants, rs13078807, rs13107325, and rs3810291 were validated in the follow-up stage [7] and rs17806313 was only significant in females [17] (Table 2).

For WHRadjBMI, both *S*_*Hom*_ and *S*_*Het*_ identified rs9864077, which was missed by conventional meta-analysis. *S*_*Het*_ identified two additional loci (rs4684854, rs2301573, Table 2 and Fig 2C). Both rs4684854 and rs2301573 were not genome-wide significant in the combined analysis of discovery and replication phases [8], but they were genome-wide significant in females only (*P* = 2.36 × 10^−08^ and *P* = 9.93 × 10^−11^, respectively) (Table 2) [17].

**Fig 3:**
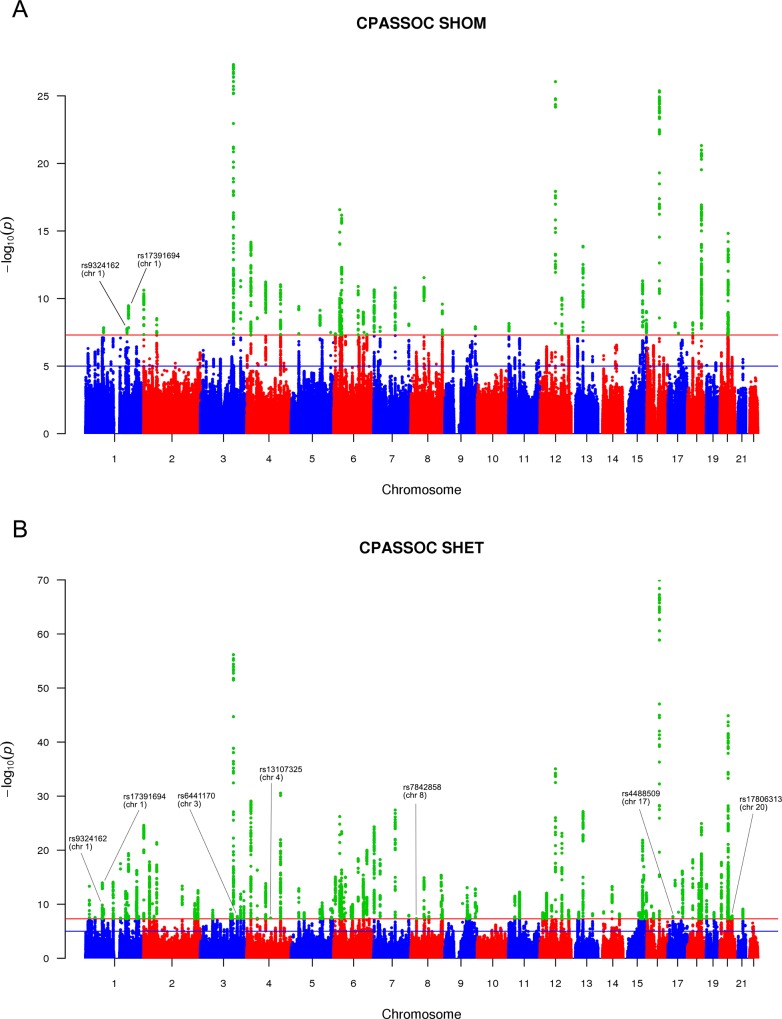
Manhattan plots of *S*_*Hom*_ and *S*_*Het*_ for combining three gender specific traits. The loci reaching genome-wide significance by *S*_*Hom*_ and *S*_*Het*_ but not by the conventional meta-analysis are marked with corresponding SNP names. (A) *S*_*Hom*_ (B) *S*_*Het*_

**Fig 4:**
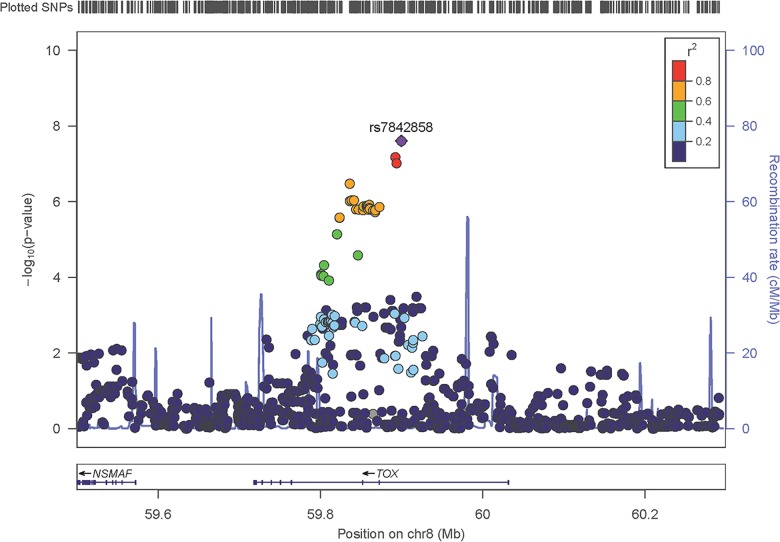
Regional plots of the novel locus rs7842858 identified by *S*_*Het*_.

When combining three gender specific traits, 7 independent variants were identified by *S*_*Hom*_ and *S*_*Het*_ but were missed by the conventional meta-analysis (Table 3 and Fig 3). Variants rs9324162 and rs17391694 were detected by both *S*_*Hom*_ and *S*_*Het*_ and were located more than 500 kb apart. These two variants were in weak linkage disequilibrium (r^2^ = 0.4). Thus, we consider them as two separate signals. Variant rs17391694 was reported to be associated with height by the GIANT study using conventional meta-analysis when combining discovery and follow-up phase data [6] [17]. The remaining five variants in Table 3 were only identified by *S*_*Het*_. SNP rs6441170 as well as rs13107325 were reported in later GIANT studies for height [9] and BMI [10], respectively. Variant rs17806313 was significantly associated with BMI in females only (Table 3). SNP rs4488509 is in high linkage disequilibrium (r^2^ = 0.84) with reported SNP rs4640244 in later GIANT studies for height [6], and they are located 450 bp away. Variant rs7842858, has not been reported previously. SNP rs7842858 is located on *TOX* (Fig 4), which has been reported to be associated with obesity and diabetes [20].

**Table 3:** Genome-wide significant loci that were only detected by CPASSOC when combining 3 traits but not by conventional meta-analysis of GIANT consortium from discovery phase.

					CPASSOC		Conventional Meta-analysis of GIANT consortium			Sex-specific Meta-analysis of GIANT consortium					
							Height	BMI	WHRadjBMI	Height		BMI		WHRadjBMI	
SNP	Chr	Position (bp)	Effect / other allele	Frequency (effect allele)	*P*-value	*P*-value	*P*-value	*P*-value	*P*-value	*P*-value (Males)	*P*-value (Females)	*P*-value (Males)	*P*-value (Females)	*P*-value (Males)	*P*-value (Females)
rs9324162	1	77489119	A/G	0.84	1.56E-08	7.97E-10	3.52E-06	3.51E-05	0.47	2.94E-05	1.5E-03	1.8E-03	2.7E-03	0.63	0.11
rs17391694	1	78157942	T/C	0.12	1.47E-08	4.51E-09	5.92E-07	1.52E-04	0.38	3.91E-05	5.2E-04	0.012	1.1E-03	0.85	0.35
rs6441170	3	158098171	T/C	0.65	0.061	3.22E-08	4.87E-07	0.0011	0.19	5.9E-04	1.11E-05	0.019	0.084	0.11	0.87
rs13107325	4	102267552	T/C	0.12	0.33	3.49E-08	2.5E-03	1.37E-07	0.16	0.016	0.02	1.1E-05	1.6E-04	0.1	0.73
rs7842858^a^	8	58987111	T/C	0.64	6.95E-05	2.49E-08	2.33E-06	0.667	0.02	3.1E-03	1E-04	6.9E-03	0.041	0.18	0.047
rs4488509	17	21381361	A/T	0.44	0.42	1.94E-08	5.33E-07	7.63E-04	0.13	1.55E-05	1.4E-04	0.016	4.1E-03	0.2	0.48
rs17806313	20	52478024	A/G	0.73	2.06E-04	1.48E-08	0.609	1.07E-05	0.27	0.095	0.41	0.49	3.5E-09	0.023	0.53

^a^New locus that has not been reported previously.

**Fig 5:**
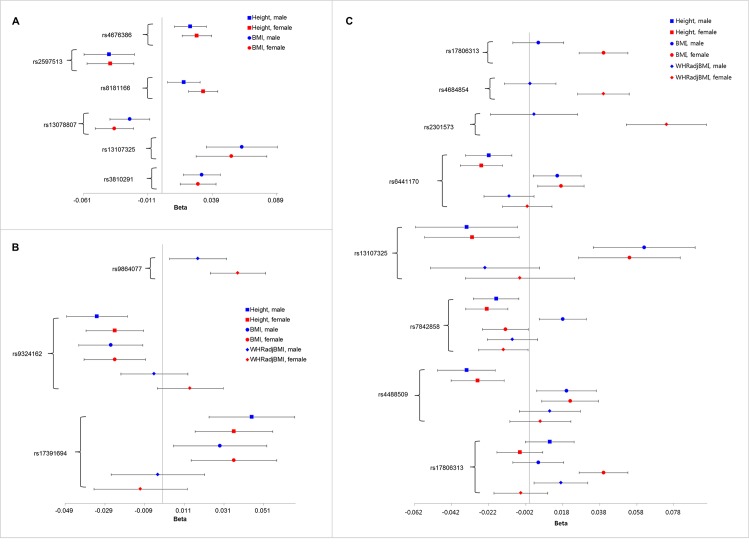
The forest plots of the effect sizes for the SNPs presented in Table 2 and 3. (A): variants only detected by *S*_*Hom*_; (B): variants detected by both *S*_*Hom*_ and *S*_*Het*_; (C) variants only detected by *S*_*Het*_ (right panel).

To compare the variants identified by *S*_*Hom*_ and *S*_*Het*_, we plotted the forest plots of the effect sizes for the SNPs in Tables 2 and 3, which were missed by the conventional meta-analysis in GIANT. We observed that the effect sizes were similar for variants only detected by *S*_*Hom*_ (Fig 5A, rs4676386, rs2597513, rs8181166, rs13078807, rs13107325, and rs3810291). All the variants detected by both *S*_*Hom*_ and *S*_*Het*_ showed some degree of heterogeneity (Fig 5B, rs9864077, rs9324162, and rs17391694). The variants detected only by *S*_*het*_ showed large amount of heterogeneity (Fig 5C, right panel of SNPs). In particularly, variants rs17806313, rs4684854 and rs2301573 only have genetic effects in females for BMI and WHRadjBMI. Variants rs644170 and rs13107325 have positive effects on BMI but negative effects on height and WHRadjBMI. Variant rs7842858 has a negative effect on height and WHRadjBMI in both genders, negative effect on BMI in males, and positive effect on BMI in females (Fig 5). The effect of rs4488509 is negative for height and positive for BMI and WHRadjBMI. The effect of rs17806313 shows substantial heterogeneity between males and females in each of the three traits.

## Discussion

In this study, we performed CPASSOC analysis using the summary statistics available from a GIANT consortium study [17]. Our results showed that CPASSOC was able to identify most of the loci detected by conventional meta-analysis of GIANT consortium. CPASSOC was also able to identify 17 loci reaching genome-wide significance (*P* < 5 × 10^−8^) that were missed by conventional meta-analysis for a single trait analysis when using the same discovery phase data. All 10 loci (Table 2) detected by CPASSOC that combine the effects of males and females for height and BMI have been replicated by GIANT using either additional replication samples or are significant in either males or female [6, 7, 11]. Our results showed that CPASSOC analysis can improve statistical power over conventional meta-analysis by combining males and females for individual traits.

When combining male and female summary statistics for height, the statistic *S*_*Het*_ identified fewer loci than either *S*_*Hom*_ or the conventional meta-analysis method, suggesting that there is rare heterogeneity between males and females for height. However, the current study only analyzed autosomal variants rather than variants on the sex chromosome.

When combining the three traits, *S*_*Hom*_ identified 55 loci while *S*_*Het*_ identified 129 loci. The substantial fewer number of variants detected by *S*_*Hom*_ suggests that many variants have different effect directions in the three traits; therefore, the association evidence by *S*_*Hom*_ is diluted. This result further indicates that the statistic *S*_*Hom*_ would be less powerful when combining multiple different traits, where substantial heterogeneity exists (Fig 5). In our analysis of combining height, BMI and WHRadjBMI, CPASSOC was able to identify additional 7 loci (Table 3) that were missed by conventional meta-analysis (Table 3 and Fig 3). Five variants (rs9324162, rs17391694, rs6441170, 13107325, and rs17806313) have been reported to be associated with height, BMI or in gender-specific analysis by the GIANT consortium using additional data [6, 9, 10, 21]. CPASSOC also identified one novel locus (rs7842858) that has not been reported to be associated with any of the three traits previously. This variant shows substantial heterogeneity among the three traits. In particular, variant rs7842858 has a positive effect on the three traits in males but negative effect in females (Fig 5), which leads to the failure of detection by either conventional single trait meta-analysis or combining the three traits by *S*_*Hom*_. SNP rs7842858 (8:58987111) resides within *TOX* (Fig 4), which has been reported to be associated with Type 2 Diabetes in Chinese Han population [20]. It is possible that this variant affects obesity variation and therefore indirectly contributes to Type 2 Diabetes. However, this hypothesis requires further studies. The CPASSOC analysis of combining height, BMI and WHRadjBMI also demonstrated pleotropic effects for all 7 loci (Fig 5). This is because the association evidence of these loci could not be detected by single trait analysis using the GIANT discovery data. The association evidence could only be detected when combining the three traits. We also observed substantial genetic heterogeneity among these three traits. When pleotropic effects exist, CPASSOC analyses of multiple traits clearly improve statistical power to detect these genetic effects. In particular, statistic *S*_*Het*_ is able to detect many variants with pleotropic effects; however, it may lose power when there is no heterogeneity, as observed in single trait analysis.

Our analysis applied a significance level 5 × 10^−8^, which is widely used in GWAS including the original GIANT publications. This criterion makes the results identified by CPASSOC comparable with that from GIANT results. Alternatively, the methods by [22], which requires genotype data, may result better genome-wide significance level. Since we only analyzed the summary statistics obtained from GIANT website, we did not applied the method by [22]. When we used a genome-wide significance level 1.0 × 10^−7^, we were able to identify additional 134 loci (S5 Table). These variants should be further examined in independent studies.

The CPASSOC [12] was designed to combine the association evidence across multiple, sometimes seemingly distinct phenotypes. Cross phenotype association will increase statistical power when analyzed traits share common variants or common genetic pathways, which may reflect the relevance of pleiotropy [4]. Thus, cross phenotype association analysis is preferred when a genetic variant affects more than one trait either through biological mediated pleiotropy. For example, in genetic study of hypertension, different measurements of blood pressure such as systolic, diastolic blood pressure, and hypertension status can be analyzed using CPASSOC [12].The hypertension related traits can be analyzed together with cardiovascular traits because of sharing potential common pathways. In the current study, we analyzed the anthropometric traits by cross phenotype analysis because these traits are likely share common biological pathways. Many traits have demonstrates different biology between males and females [17]. Sex-specific analysis is able to detect specific variants to a sex group and therefore such analysis should always be performed. On the other hand, males and female share common biology and combined analysis will increase a study sample size and consequently increase statistical power for identifying variants shared by both males and females. Our results show that CPASSOC analysis can detect sex-specific variants as well as the variants shared by both sexes.

We also observed much larger correlations between male and female summary statistics for height and BMI compared to that of WHRadjBMI (S2 Table), although we pruned SNPs with linkage disequilibrium and excluded SNPs with large summary statistics. These high correlations for height and BMI may not be surprising because of the contributions of many genetic variants with small effect sizes for both traits. In comparison, the lower correlation between male and female summary statistics for WHRadjBMI may suggest that most of genetic contribution is shared between BMI and WHR.

There are several recently developed statistical approaches in analyzing multiple traits at summary statistics level, including the ASSET [23] and the Cross Phenotype Meta-Analysis (CPMA) [24]. The ASSET is suitable for identifying a subset of associated traits while CPASSOC directly evaluates the aggregated association evidence between a SNP to multiple phenotypes. CPASSOC is much faster than the ASSET computationally because ASSET has to search all possible subsets. When the number of traits and studies increased, the number of possible subsets can grow exponentially. The Cross Phenotype Meta-Analysis (CPMA) was developed to test whether there is association of a SNP to multiple phenotypes, or a true pleiotropy effect. When one of traits is not associated with a SNP, CPMA will not have statistical power. It will be interesting to perform these two tests to search for the subsets of traits associated with a SNP or to test for pleiotropy effect in the GIANT consortium.

In conclusion, CPASSOC identified 17 loci associated with anthropometric traits that were missed by conventional meta-analysis for a single anthropometric trait in GIANT consortium studies that used the same discovery phase data as we did. CPASSOC is also able to detect pleiotropic effects when analyzing multiple traits.

## Materials and Methods

### Datasets

The summary statistics of height, BMI and WHRadjBMI were downloaded from the GIANT consortium website (https://www.broadinstitute.org/collaboration/giant/index.php/GIANT_consortium_data_files). The downloaded data include sex-specific [17] and sex-combined [6–8] GWASs meta-analysis summary statistics from the discovery phase. For discovery stage in sex- specific studies, 46 studies (up to 60,586 males, 73,137 females) on height and BMI and 32 studies (up to 34,629 males, 42,969 females) on WHR were included [17]. In the discovery phase of sex-combined studies, summary statistics were collected from 46 GWASs in a meta-analysis of 133,653 individuals (60,587 males and 73,066 females) for height [6], 46 studies with up to 123,865 individuals for BMI [7], and 32 studies with up to 77,167 individuals (34,601 males and 42,735 females) for WHRadjBMI [8].

### Cross-phenotype association analysis

We applied the CPASSOC package developed by Zhu et al. [12] to combine association evidence of both sexes with height, BMI and WHRadjBMI. CPASSOC can integrate association evidence from summary statistics of multiple traits. It uses summary-level data from single SNP-trait association of GWASs to detect which variant is associated with at least one trait. This method improves statistical power by analyzing multiple phenotypes and it can be executed with the summary statistics from GWASs. CPASSOC provides two statistics, *S*_*Hom*_ and *S*_*Het*_. *S*_*Hom*_ is similar to the fixed effect meta-analysis method [25] but accounting for the correlation of summary statistics among cohorts induced by potential overlapped or related samples. In brief, assuming we have summary statistical results of GWAS from *J* cohorts with *K* phenotypic traits. In each cohort, single SNP-trait association was analyzed for each trait separately. Let *T*_*jk*_ be a summary statistic for a SNP, *j*^*th*^ cohort and *k*^*th*^ trait. Let *T* = (*T*_11_,⋯,*T*_*J*1_, ⋯,*T*_1*K*_,⋯, *T*_*JK*_)^*T*^ represents a vector of test statistics for testing the association of a SNP with *K* traits. We used a Wald test statistic $T_{jk}=\frac{{\hat{\beta }}_{jk}}{{\hat{s}}_{jk}}$, where ${\hat{\beta }}_{jk}\;and\;{\hat{s}}_{jk}$ are the estimated coefficient and corresponding standard error for the *k*^*th*^ trait in the *j*^*th*^ cohort. *S*_*Hom*_ is then defined as

$$S_{Hom}=\frac{e^{T}({RW)}^{-1}T{\left(e^{T}({RW)}^{-1}T\right)}^{T}}{e^{T}(WR{W)}^{-1}e},$$ (1)

which follows a *χ*^2^ distribution with one degree of freedom, where *e*^*T*^ = (1,…,1) has length *J* × *K* and *W* is a diagonal matrix of weights for the individual test statistics. We used the sample sizes for the weights, i.e., $w_{jk}=\sqrt{n_{j}}$ for the sample size *n*_*j*_ of the *j*^*t*h^ cohort.

To define *S*_*Het*,_ we first let

$$S(\tau )=\frac{e^{T}({R(\tau )W(\tau ))}^{-1}T(\tau ){\left(e^{T}({R(\tau )W(\tau ))}^{-1}T(\tau )\right)}^{T}}{e^{T}{{W(\tau )}^{-1}R(\tau )}^{-1}{W(\tau )}^{-1}e},$$

where *T*(*τ*) is the sub-vector of *T* satisfying |*T*_*jk*_| > *τ* for a given *τ*>0, and *R*(*τ*) is a sub-matrix of *R* representing the correlation matrix, and *W*(*τ*) be the diagonal submatrix of *W*, corresponding to *T*(*τ*). Here we let $w_{jk}=\sqrt{n_{j}}\times sign(T_{jk})$. Then the test statistic is *S*_*Het*_ = max_*τ*>0_*S*(*τ*).

The asymptotic distribution of *S*_*Het*_ does not follow a standard distribution but can be evaluated using simulation. *S*_*Het*_ is an extension of *S*_*Hom*_ but power can be improved when the genetic effect sizes vary for different traits. The distribution of *S*_*Het*_ under the null hypothesis can be obtained through simulations or approximated by an estimated beta distribution. We first applied both *S*_*Hom*_ and *S*_*Het*_ to combine sex-specific summary statistics for each of the three traits and compared the results with those from conventional meta-analysis of the same discovery phase data in GIANT consortium studies [6–8]. We next applied both *S*_*Hom*_ and *S*_*Het*_ for combining all the sex-specific summary statistics of the three traits: height, BMI and WHRadjBMI. We hypothesized that meta-analyzing multiple traits would allow us to identify additional variants that are likely to be missed by the conventional meta-analyses for a single trait.

To perform CPASSOC analysis, a correlation matrix is required to account for the correlation among phenotypes or induced by overlapped or related samples from different cohorts. Zhu et al. [12] suggested using a set of SNPs in linkage equilibrium to estimate the correlation coefficients. We selected the SNP set based on linkage disequilibrium (LD) pattern in the ARIC European American (EA) data (downloaded from dbGaP http://www.ncbi.nlm.nih.gov/gap). In brief, the ARIC EA cohort includes 9,707 individuals with approximately 840,000 SNPs genotyped on the Affymetrix Array 6.0 [26, 27]. We first applied pairwise LD pruning with r^2^ threshold of 0.2 using the software PLINK (http://pngu.mgh.harvard.edu/purcell/plink/). SNPs with large effect sizes may represent true association, and consequently may inflate correlation among summary statistics. Therefore, we removed SNPs whose summary statistics Z scores were greater than 1.96 or less than -1.96. The final SNP sets for correlation estimation include 81,322 SNPs for height, 82,012 SNPs for BMI, and 81,130 SNPs for WHRadjBMI. We chose the common sets of SNPs for both sexes and three traits that can be mapped to dbSNP human Build 142 to perform the CPASSOC analyses. The numbers of SNPs used in this study are presented in S1 Table.

We reported loci that reached genome-wide significance (*P* < 5 × 10^−8^) by CPASSOC from sex-specific data [17], but not by sex-combined conventional meta-analysis [6–8] when using the same samples from the discovery phase. Here we applied the same significant level *P* = 5 × 10^−8^ as in GWAS because CPASSOC performs the same number of tests although multiple traits are analyzed. To do this, for a SNP reaching *P* < 5 × 10^−8^ by either *S*_*Hom*_ or *S*_*Het*_, we examined the region within 500 kb of each side of the SNP. The SNP was considered to be identified only by CPASSOC if no SNPs that are genome-wide significant with conventional meta-analysis from the discovery phase data were found in the 1.0 Mb region, and it is not in LD with the index SNPs of the GIANT studies. We performed meta-analysis by combining male and female data for each trait separately, as well as by combining all the three traits and both sexes.

## Supporting Information

S1 Table The number of SNPs mappable to dbSNP human Build 142, which were used in meta-analysis with CPASSOC. ^a^SNPs used for conventional meta-analyses of sex-combined discovery phase data in the GIANT consortium studies. ^b^Intersection set of SNPs used for conventional meta-analyses of sex-specific discovery phase data in the GIANT consortium studies. ^c^Intersection set of SNPs between sex-combined and sex-specific study. ^d^Intersection set of SNPs among height, BMI, and WHRadjBMI.
(DOCX)

S2 Table Correlations between male and female cohort for each trait and those between combinations of sex and trait. (DOCX)

S3 Table SNPs representing identified through either conventional meta-analysis (sex combined and sex-specific) or CPASSOC for height, BMI, and WHRadjBMI. (XLSX)

S4 Table SNPs representing identified through either CPASSOC for combining 3 sex-specific traits or conventional meta-analysis of GIANT consortium (sex-combined and sex-specific) in discovery phase data. (XLSX)

S5 Table SNPs with P-values between 1E-7 and 5E-8 for either *S*_*Hom*_ or *S*_*Het*_ from CPASSOC when combining three traits. (XLSX)
