# Supplementary material for: Multivariate Analysis of Anthropometric Traits Using Summary Statistics of Genome-Wide Association Studies from GIANT Consortium
Source: PLoS One. 2016 Oct 4;11(10):e0163912. doi: 10.1371/journal.pone.0163912 (PMC5049793; doi:10.1371/journal.pone.0163912)
Supplement: S1 Table — aSNPs used for conventional meta-analyses of sex-combined discovery phase data in the GIANT consortium studies. bIntersection set of SNPs used for conventional meta-analyses of sex-specific discovery phase data in the GIANT consortium studies. cIntersection set of SNPs between sex-combined and sex-specific study. dIntersection set of SNPs among height, BMI, and WHRadjBMI. (DOCX) [file pone.0163912.s001.docx]

S1 Table. The number of SNPs mappable to dbSNP human Build 142, which were used in meta-analysis with CPASSOC.

| Trait | Sex-combined study^a^ | Sex-specific study^b^ | Sex-combined study ∩ sex-specific study^c^ |
| --- | --- | --- | --- |
| Height | 2,465,409 | 2,736,584 | 2,465,002 |
| BMI | 2,467,273 | 2,733,958 | 2,466,863 |
| WHRadjBMI | 2,443,083 | 2,725,322 | 2,442,484 |
| Common for 3 traits^d^ | 2,432,631 | 2,723,278 | 2,432,243 |

^a^SNPs used for conventional meta-analyses of sex-combined discovery phase data in the GIANT consortium studies [6 - 8].

^b^Intersection set of SNPs used for conventional meta-analyses of sex-specific discovery phase data in the GIANT consortium studies [17].

^c^Intersection set of SNPs between sex-combined and sex-specific study.

^d^Intersection set of SNPs among height, BMI, and WHRadjBMI.
