# Supplementary material for: Multivariate Analysis of Anthropometric Traits Using Summary Statistics of Genome-Wide Association Studies from GIANT Consortium
Source: PLoS One. 2016 Oct 4;11(10):e0163912. doi: 10.1371/journal.pone.0163912 (PMC5049793; doi:10.1371/journal.pone.0163912)
Supplement: S2 Table — (DOCX) [file pone.0163912.s002.docx]

**S2 Table. Correlations between male and female cohort for each trait and those between combinations of sex and trait. SNPs were selected base on LD pruning with R2=0.20 in ARIC European data. SNPs with absolute Z score larger than 1.96 were excluded.**

|  |  | Height | | BMI | | WHRadjBMI | |
| --- | --- | --- | --- | --- | --- | --- | --- |
|  |  | Male | Female | Male | Female | Male | Female |
| Height | Male | 1 | 0.128 | -0.038 | -0.007 | -0.004 | -0.002 |
|  | Female | 0.128 | 1 | -0.012 | -0.056 | 0.007 | -0.003 |
| BMI | Male | -0.038 | -0.012 | 1 | 0.096 | 0.010 | -0.007 |
|  | Female | -0.007 | -0.056 | 0.096 | 1 | 0.011 | 0.001 |
| WHRadjBMI | Male | -0.004 | 0.007 | 0.010 | 0.011 | 1 | 0.031 |
|  | Female | -0.002 | -0.003 | -0.007 | 0.001 | 0.031 | 1 |
